# Tween 80 Micelles Loaded with Fe_3_O_4_ Nanoparticles and Artemisinin for Combined Oxygen-Independent Ferroptosis Therapy of Cancer

**DOI:** 10.3390/pharmaceutics16050639

**Published:** 2024-05-09

**Authors:** Junming Cui, Xinxi Cai, Rui Qian, Lin Wu, Xueyong Qi, Jin Cao, Song Shen

**Affiliations:** 1 Department of Pharmacy, Affiliated Hospital of Jiangsu University, Zhenjiang 212001, China; 13730914511@163.com (J.C.); caixinxi111@163.com (X.C.); qianruiemail@163.com (R.Q.); 2 College of Pharmaceutical Sciences, Jiangsu University, Zhenjiang 212013, China; qixyemail@163.com (X.Q.); caojin0830126@163.com (J.C.)

**Keywords:** artemisinin, iron oxide nanoparticles, tween 80, micelles, cancer therapy

## Abstract

Artemisinin has an endoperoxide bridge structure, which can be cleaved by ferrous ions to generate various carbonyl radicals in an oxygen-independent manner, highlighting its potential for treating hypoxic tumors. In our study, we fabricated Tween 80 micelles loaded with Fe_3_O_4_ nanoparticles and artemisinin for cancer therapy. The synthesized Fe_3_O_4_ nanoparticles and drug-loaded micelles have particle sizes of about 5 nm and 80 nm, respectively, both exhibiting excellent dispersibility and stability. After uptake by MCF-7 cells, drug-loaded micelles release Fe^2+^ and ART into the cytoplasm, effectively inducing the generation of reactive oxygen species (ROS) in hypoxic conditions, thereby enhancing toxicity against cancer cells. In vitro and in vivo studies have demonstrated that ART and Fe_3_O_4_ nanoparticles are encapsulated in Tween 80 to form micelles, which effectively prevent premature release during circulation in the body. Although free ART and Fe_3_O_4_ nanoparticles can inhibit tumor growth, TW80-Fe_3_O_4_-ART micelles demonstrate a more pronounced inhibitory effect, with a tumor suppression rate of up to 85%. A novel strategy based on artemisinin and ferroptosis is thus offered, holding a favorable prospect for hypoxic cancer therapy.

## 1. Introduction

Ferroptosis is a form of cell death characterized by iron-dependent reactive oxygen species (ROS) induction and lipid peroxidation [1,2,3]. It is distinct from forms such as apoptosis, necrosis, and autophagy. When an excess of ferrous ions is presented in the cells, they will catalyze the conversion of H_2_O_2_ through the Fenton (Fe^2+^ + H_2_O_2_ → Fe^3+^ + OH^−^ + ·OH) and Haber-Weiss reactions to produce highly reactive hydroxyl radicals (·OH) [4,5]. Generally, ferroptosis is oxygen-dependent since the presence of oxygen can enhance the generation of ROS and lipid peroxides [6]. However, the hypoxic environment in the tumor weakened the therapeutic effect of ferroptosis [7]. Fe_3_O_4_ nanoparticles (NPs) can induce ferroptosis in tumor cells through the Fenton reaction [8]. What’s more, Fe_3_O_4_ NPs of different sizes exhibit distinct anti-tumor effects. On the one hand, Fe_3_O_4_ NPs with a larger diameter (50–200 nm) can accumulate significantly at tumor sites due to the enhanced permeation and retention (EPR) effect, yet they exhibit limited intratumoral penetration [9]. On the other hand, ultra-small iron oxide nanoparticles display optimal tissue penetration but are susceptible to metabolic clearance [9,10].

Artemisinin (ART), which is a sesquiterpene lactone compound featured by the distinctive endoperoxide bridge (-O-O-), is a potent drug renowned for its remarkable effectiveness against malaria [11,12]. In recent years, research has indicated that ART also demonstrates promising efficacy in killing tumor cells [13,14]. In comparison to normal cells, tumor cells not only exhibit an enhanced ability to synthesize ferroheme but also maintain elevated intracellular iron concentrations to sustain their uncontrolled proliferation. Existing research indicates that the biological activity of ART relies on the activation of iron (II), rendering it selectively advantageous for targeting tumor cells over normal cells [14,15,16,17]. About the iron-dependent bioactivation of ART under a hypoxic environment, the currently widely accepted model for the reduction and cleavage of the ART endoperoxide bridge involves the binding of a low-valence transition metal ion (either exogenous Fe^2+^ or ferroheme) to ART. The endoperoxide bridge of the ART can be cleaved in the presence of Fe^2+^ due to the catalyzation effect of the low-valence transition metal ions to form initial carbon-centered free radicals or subsequent carbon-centered free radicals, which can damage the cancer cells in an oxygen-independent manner [15,18,19,20]. Meanwhile, ART induces lysosomal degradation of ferritin, which is a pool to store the excess iron [21,22]. Exposure of cancer cells to ART leads to reduced proliferation, elevated oxidative stress levels, induction of apoptosis, and inhibition of angiogenesis. These effects are primarily attributed to the release of iron (II)-mediated reactive oxygen species (ROS) or carbon-centered free radicals.

In this work, we synthesized ultrasmall hydrophobic Fe_3_O_4_ nanoparticles and then assembled the Fe_3_O_4_ nanoparticles into the core of the polysorbate 80 (Tween-80, TW-80) to form magnetic micelles. The micelles possess the following characteristics. (1) Due to particle size, the micelles can be passively targeted to the tumor site, achieving enrichment; (2) ART and Fe^2+^/Fe^3+^ donors can be co-transported to the tumor site for release; (3) The micelles can induce the generation of ROS and carbonyl free radicals, enhancing the efficacy of ferroptosis.

## 2. Materials and Methods

### 2.1. Materials and Reagents

Iron (III) acetylacetonate (Fe(acac)_3_), 1,2-hexadecaendiol, dibenzyl ether, oleylamine, oleic acid, anhydrous ethanol, chloroform, tween 80 (TW80), 2, 2′-bipyridine, hydrogen peroxide (H_2_O_2_), dimethyl sulfoxide (DMSO), sodium hydroxide (NaOH) were purchased from Sinopharm Chemical Reagents Co., Ltd. (Shanghai, China). Coumarin-3-carboxylic acid (3-CCA), 3-(4, 5-dimethylthiazol-2-yl)-2, 5-diphenyltetrazolium bromide (MTT), 2, 2′-azino-bis (3-ethylbenzothiazoline-6-sulfonic acid) (ABTS), Hoechst 33342, Lysogreen, doxorubicin hydrochloride (DOX), and paraformaldehyde were purchased from Sigma-Aldrich Co. (Shanghai, China). Indocyanine green (ICG), 2, 7-Dichlorodihydrofluorescein diacetate (DCFH-DA) and artemisinin (ART) were purchased from Aladdin Reagent Co., Ltd. (Shanghai, China). All reagents were of analytical purity and used without further purification. The water used in all experiments was deionized with a Millipore Milli-Q system.

### 2.2. Synthesis of Fe_3_O_4_ NPs

The synthesis of ultra-small diameter Fe_3_O_4_ nanoparticles was prepared using an organic solvent for the thermal decomposition [23,24]. The 0.706 g of Fe(acac)_3_, 2.584 g of 1,2-hexadecaendiol, 1 mL of oleylamine, and 1 mL of oleic acid were mixed with 20 mL dibenzyl ether in a breaker and then stirred for 20 min at 80 °C to dissolve. The reaction was conducted under a nitrogen atmosphere to prevent oxidation. Subsequently, the mixture was heated to 200 °C for 1.5 h, followed by heating at 260 °C for half an hour to induce the nucleation and growth of Fe_3_O_4_ NPs. After the reaction was completed, the product cooled to room temperature and purified by washing it with ethanol to remove the organic solvent. Finally, the synthetic Fe_3_O_4_ NPs were collected via magnet separation and dispersed in ethanol for preservation.

### 2.3. Preparation of TW80-Fe_3_O_4_-ART Micelles

To prepare TW80-Fe_3_O_4_-ART micelles, 500 μg Fe_3_O_4_ nanoparticles were dispersed in 0.2 mL chloroform under stirring, and 20 mg ART was dissolved in the obtained solution. Then, the mixture solution was slowly introduced in drops into a 10 mL aqueous solution containing 150 mg TW80 under magnetic stirring for 30 min. After centrifugation at 12,000 rpm for 10 min, the precipitates were collected and washed with water. Subsequently, the prepared TW80-Fe_3_O_4_-ART micelles were freeze-dried to obtain a solid for further use and preservation.

### 2.4. Characterization

The morphologies of Fe_3_O_4_ NPs and TW80-Fe_3_O_4_-ART micelles were characterized by transmission electron microscopy (Tecnai-12, Philips, Eindhoven, Holland). The particle size distribution of TW80-Fe_3_O_4_-ART micelles in an aqueous solution was determined by the dynamic light scattering method (MS-3000, Malvern Instruments, Malvern, UK) at room temperature. The FT-IR spectra of ART, Fe_3_O_4_ NPs, TW80, and TW80-Fe_3_O_4_-ART were characterized by a Fourier Transform Infrared Spectrometer (FTIR-370, Nicolet Avatar, Madison, WI, USA).

### 2.5. Detection of Fe^2+^ Release and Hydroxyl Radical (·OH) from TW80-Fe_3_O_4_-ART Micelles

Fe^2+^ release was detected by 2, 2′-bipyridine [25]. Place 1 mL of TW80-Fe_3_O_4_-ART micelles (5 mg/mL) into 1 mL of phosphate-buffered saline (PBS) with pH values of 7.4, 6.0, and 5.0, respectively. Add 2 mL of 2, 2′-bipyridine with a concentration of 200 μM. After a 12-h reaction, record the absorbance at 480 nm (UV-2450, Shimadzu, Kyoto, Japan).

In our study, we utilized 3-CCA (λ_ex_ = 388 nm, λ_em_ = 450 nm) to detect the generation of ·OH during the catalytic Fenton reaction of the material [26]. TW80-Fe_3_O_4_-ART micelles (0.5 mL, 5 mg/mL) were placed in 1.5 mL of PBS with pH values of 7.4, 6.0, and 5.0, respectively. After 12 h of incubation, H_2_O_2_ (50 μL, 1 M) and 3-CCA (200 μL, 1 mM) were added to the supernatant, followed by a 30-min photophobic reaction. The fluorescence intensity was measured by the Fluorescence Spectrophotometer (RF-6000, Shimadzu, Kyoto, Japan).

### 2.6. Free-Radical Detection in Solution

The ROS generated by the reaction between ART and Fe^2+^ can be detected using ABTS [27]. A mixture of ABTS aqueous solution (2 mg/mL, 1 mL) and TW80-Fe_3_O_4_-ART micelles aqueous solution (10 mg/mL, 1 mL) at different pH values (7.4, 6.0, and 5.0) was prepared and maintained in darkness at 37 °C for 12 h. Following centrifugation, the supernatant was collected to measure the absorbance of the ABTS solution within the 600~850 nm range.

### 2.7. Release of ART from TW80-Fe_3_O_4_-ART Micelles

The release kinetics of ART from the micelles were studied in PBS solution (pH = 7.4, 6.0, and 5.0) containing 10% ethanol for 6 h. In short, 5 mL TW80-Fe_3_O_4_-ART dispersion (1 mg/mL) was subjected to light-shielding treatment by covering with tin foil and placed in a shaking incubator at 37 °C with a constant agitation speed of 180 rpm. At discrete time intervals (0, 1, 2, 3, 4, 5, and 6 h), the samples underwent centrifugation at 10,000 rpm for 10 min, followed by a collection of 2 mL of the supernatant. Subsequently, an equal volume of PBS was added, and the mixture was subjected to continued agitation. The ART supernatant was hydrolyzed with NaOH at 25 °C for 30 min, and the absorbance was measured at 292 nm.

### 2.8. Intracellular Free-Radical Detection

MCF-7 cells (1 × 10^5^ cells/well) were seeded in six-well cell culture plates for 24 h. Cells were incubated with TW80, Fe_3_O_4_ NPs, ART, and TW80-Fe_3_O_4_-ART micelles with or without oxygen for 6 h. Cell nuclei were stained with Hoechst 33342 (10 μg/mL, 1 mL, 15 min), and the DCFH-DA (1 mL, 10 µM) method was carried out to detect intracellular free radical level [28]. After being washed three times with PBS, the cells were fixed with a 4% paraformaldehyde solution and subsequently examined under an inverted fluorescence microscope (DSY2000X, Leica, Heidelberg, Germany).

### 2.9. Cell Uptake

To evaluate the cellular uptake of TW80-Fe_3_O_4_-ART micelles, MCF-7 cells were cultured in glass-bottom cell culture dishes for 24 h. Subsequently, the cells were exposed to both free DOX and DOX-labeled TW80-Fe_3_O_4_-ART micelles at equivalent DOX concentrations (10 µg/mL) for either 1 or 4 h. Cytoplasmic and nuclear staining was performed using Lysogreen (2 μM) and Hoechst 33342 (10 μg/mL), respectively. Following a single wash with PBS, the uptake patterns were visualized using confocal laser scanning microscopy (Delta Vision TM Elite, General Electric, Boston, MA, USA).

### 2.10. In Vitro Cytotoxicity Test

To assess the cytotoxicity, MCF-7 cells were incubated with TW80, ART, Fe_3_O_4_ NPs, or TW80-Fe_3_O_4_-ART micelles for 24 h. After removing non-internalized nano drugs using PBS, 100 μL of MTT (1 mg/mL) was dispensed into each well. Incubation was carried out at 37 °C for 4 h. Subsequently, 150 μL of DMSO was added to each well to dissolve the MTT-reduced water-soluble formazan crystals. Finally, absorbance was measured using a microplate reader (Spectra Max 190, Molecular Devices, Silicon Valley, CA, USA) at a wavelength of 570 nm.

### 2.11. Intracellular ·OH Detection

MCF-7 cells were seeded in glass bottom cell culture dishes for 24 h. After incubating MCF-7 cells with 1 mL ART, Fe_3_O_4_ NPs, and TW80-Fe_3_O_4_-ART micelles for 2 h and 6 h, the cells were incubated with 10 μM 3-CCA for 45 min and then stained with 1 mL Lysogreen (2 μM) for 45 min. The fluorescence images of the cells were obtained by confocal laser scanning microscopy.

### 2.12. Animal Experiment

Male ICR mice aged 6 weeks were purchased from Jiangsu University Laboratory Animal Center (Zhenjiang, Jiangsu, China, KY-20230127). All animal procedures were performed under the protocols approved by the Institutional Animal Care and Use Committee of Jiangsu University Laboratory Animal Center (approved date: 2023-06-14). All mice were individually housed in ventilated cages maintained at a temperature of 21 ± 2 °C and a relative humidity of 55 ± 10%, under a 12-h light-dark cycle in aseptic conditions. Laboratory feed and water were provided to all mice. The mice entered the experimental phase after one week of acclimatization.

S180 (mouse ascites tumor cell line) tumor cells were intraperitoneally injected into several male ICR mice to culture S180 tumor cells. Then, cultured S180 tumor cells (2 × 10^6^) were injected subcutaneously into the armpit of the right forelimb of male ICR mice to establish an S180 sarcoma-bearing mouse model. After 7–10 days of cultivation, the model was successfully established.

### 2.13. Tissue Distribution Studies

Free indocyanine green (ICG) and ICG-labeled TW80-Fe_3_O_4_-ART micelles were intravenously administered to S180 tumor-bearing mice (2 mg/kg, based on ICG concentration) [29]. The mice were euthanized at predetermined time points (5 h, 10 h, 24 h, and 48 h), and various organs were excised for ex vivo organ fluorescence imaging to investigate the pharmacokinetic distribution of the prepared micelles in the animal model, with free ICG used as the control. Results were recorded using the in vivo imaging system (Maestro TM, CRI, USA).

### 2.14. In Vivo Therapy

S180 tumor-bearing mice were randomly allocated into five groups, each comprising six mice (n = 6): (a) PBS, (b) TW80, (c) Free ART, (d) Fe_3_O_4_ NPs, (e) TW80-Fe_3_O_4_-ART micelles. All formulations were administered intravenously through the tail vein at three-day intervals during a 15-day treatment regimen. The body weights were monitored on days 0, 3, 6, 9, 12, and 15. The dosages administered to the various groups were 5 mg/kg of ART or Fe_3_O_4_ NPs. For TW80-Fe_3_O_4_-ART micelles group, the dosage of ART was 5 mg/kg. After 15 days of treatment, euthanasia was performed on all mice, followed by the extraction of their hearts, livers, spleens, lungs, kidneys, and tumors. Tissues were then fixed in 4% paraformaldehyde and subsequently embedded in paraffin wax. The resulting tissue sections were stained using hematoxylin and eosin (H&E) as well as Prussian blue staining techniques.

### 2.15. Statistical Analysis

The data were analyzed statistically and presented as a mean ± standard deviation (SD) from at least 3 independent experiments. One-way ANOVA analyses were processed with SPSS Statistics 27. The significance of differences is handled as follows: ns, not significant, * *p* < 0.05, ** *p* < 0.01, *** *p* < 0.001.

## 3. Results

### 3.1. Characterization of Fe_3_O_4_ NPs and TW80-Fe_3_O_4_-ART Micelles

**Figure 1:**
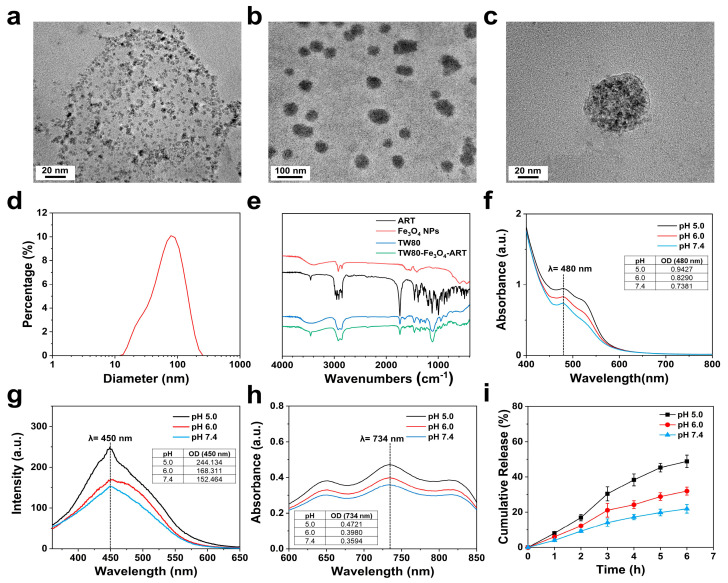
TEM of the (**a**) Fe_3_O_4_ NPs, (**b**,**c**) TW80-Fe_3_O_4_-ART micelles with different scales. (**d**) Size distribution of TW80-Fe_3_O_4_-ART micelles. (**e**) The FTIR spectra of ART, Fe_3_O_4_ NPs, TW80 and TW80-Fe_3_O_4_-ART micelles. (**f**) Fe^2+^ release characteristic of TW80-Fe_3_O_4_-ART micelles. (**g**) The fluorescence of 3-CCA in the presence of TW80-Fe_3_O_4_-ART micelles. (**h**) Absorption of ABTS^+·^ showing the free radicals produced by TW80-Fe_3_O_4_-ART micelles. (**i**) In vitro release of ART from TW80-Fe_3_O_4_-ART micelles.

Fe_3_O_4_ NPs were synthesized using a high-temperature thermal decomposition method with Fe(acac)_3_ as the precursor. Then, the Fe_3_O_4_ nanoparticles were assembled into TW80 micelles. The morphology of Fe_3_O_4_ NPs and TW80-Fe_3_O_4_-ART micelles was characterized by transmission electron microscopy (TEM). As shown in Figure 1a–c, Fe_3_O_4_ NPs and TW80-Fe_3_O_4_-ART micelles displayed a spheroidal shape with average diameters of about 5 nm and 80 nm, respectively. As depicted in Figure 1d, the hydrated particle size of TW80-Fe_3_O_4_-ART micelles was detected to be about 90 nm owing to the hydrated sheath. In Figure 1e, it could be observed in the absorption spectrum of TW80-Fe_3_O_4_-ART that there were noticeable absorption peaks of Fe_3_O_4_ NPs and ART, indicating the successful loading of Fe_3_O_4_ NPs and ART. The drug loading and encapsulation efficiency of ART were determined to be 7.9% and 67.5%, respectively.

### 3.2. Detection of Fe^2+^ Release and ROS Generation

In general, intracellular iron-based nanoparticles can release either Fe^2+^ or Fe^3+^. The release of Fe^2+^ can induce the Fenton reaction within cells, leading to the generation of highly oxidative ·OH radicals, which in turn causes lipid peroxidation and subsequently results in cell death. In order to preliminarily demonstrate the ability of TW80-Fe_3_O_4_-ART to release Fe^2+^ in a mildly acidic environment, generate Fenton reaction-derived ·OH, and activate ART to generate ROS, we employed 2, 2′-bipyridine, 3-CCA, and ABTS as probes for detection. 2, 2′-bipyridine can react with Fe^2+^ to form complexes, generating a distinctive UV absorption peak at 480 nm. As depicted in Figure 1f, with a decrease in solution pH, the release of Fe^2+^ from TW80-Fe_3_O_4_-ART micelles increased. 3-CCA can be hydroxylated by ·OH to produce 7-hydroxycoumarin with blue fluorescence. Therefore, this effect of pH on the generation of ·OH was monitored using 3-CCA as a probe. Since the release of Fe^2+^ in TW80-Fe_3_O_4_-ART micelles is pH-dependent, we hypothesize that the generation of ·OH should also be pH-dependent. As shown in Figure 1g, TW80-Fe_3_O_4_-ART micelles exhibited the higher catalytic ability to generate ·OH radicals at a lower pH environment. This result validates this assumption. In the presence of ferrous ions, the peroxide bridge of ART cleaves, generating free radicals in an oxygen-independent manner. To demonstrate this point, ABTS can be employed for the detection of these generated free radicals. Upon free radical oxidation, ABTS is transformed into a green-colored ABTS^+·^ species, exhibiting ultraviolet absorption at 734 nm. As shown in Figure 1h, TW80-Fe_3_O_4_-ART micelles released the highest amount of ROS in a slightly acidic environment at pH 5.0. It is worth noting that, to prevent any interference from ·OH on the detection results, we did not introduce H_2_O_2_ to induce the Fenton reaction of Fe^2+^.

### 3.3. Release of ART from TW80-Fe_3_O_4_-ART Micelles

Stimuli-responded release in the tumor is preferred for drug delivery. To investigate the pH-sensitive release behavior of TW80-Fe_3_O_4_-ART, three acidity solutions were selected as external stimuli. We determined the release of ART at different pH values (pH 7.4, 6.0, and 5.0) by detecting the concentration of ART using UV-Vis spectrophotometry. As shown in Figure 1i, the accumulated release rates of TW80-Fe_3_O_4_-ART micelles at pH 7.4 and 5.0 were 27.9% and 70.8% after 6 h, indicating pH-sensitive release.

### 3.4. Intracellular Free-Radical Detection

**Figure 2:**
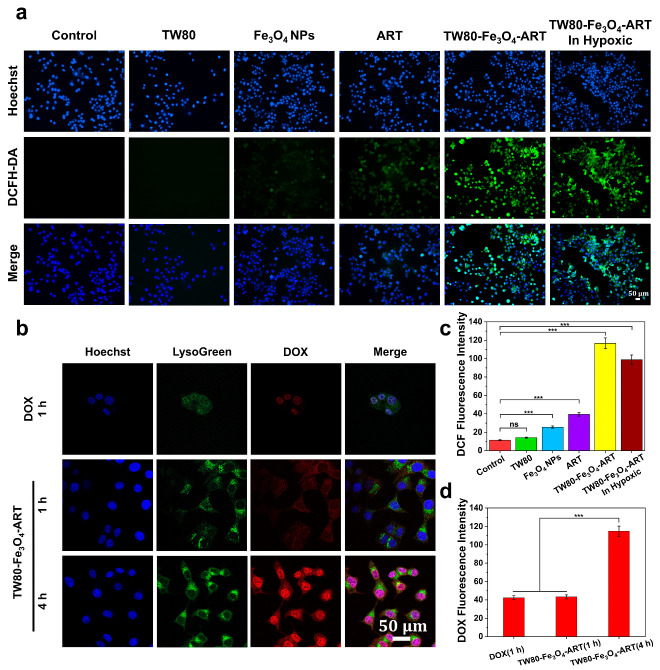
(**a**) Intracellular ROS production was monitored using DCFH-DA as a fluorescent probe. Blue: hoechst, green: DCF. (**b**) The intracellular uptake of free DOX and TW80-Fe_3_O_4_-ART micelles. Blue: hoechst, green: lysogreen, red: DOX. (**c**) Quantitative analysis of DCF fluorescence intensity was performed using the Image J software system (version 1.8.0, n = 3). (**d**) Quantitative analysis of DOX fluorescence intensity was performed using the Image J software system (n = 3). The bar graphs represent mean ± SD. ns, not significant, *** *p* < 0.001.

DCFH-DA is non-fluorescent and can be oxidized by intracellular ROS to form 2,7-dichlorofluorescein (DCF) with green fluorescence. The generation of ROS induced by TW80, Fe_3_O_4_ NPs, ART, and TW80-Fe_3_O_4_-ART micelles treatments was investigated using DCFH-DA as a probe. As shown in Figure 2a,c, a faint green fluorescence was observed in ART and Fe_3_O_4_ NPs, while the TW80-Fe_3_O_4_-ART group displayed significantly higher fluorescence. Fluorescence was scarcely observed in both the control and TW80 groups.

### 3.5. Cell Uptake

We investigated the cellular uptake of TW80-Fe_3_O_4_-ART micelles at 1 h and 4 h. As shown in Figure 2b,d, TW80-Fe_3_O_4_-ART micelles exhibited time-dependent uptake, with a higher fluorescence intensity in the 4 h. Meanwhile, we noticed that after incubation for 1 h, the red fluorescence was mainly located in the cytoplasm. However, the red fluorescence was located in the nucleus after incubation for 4 h, indicating the release of DOX from the nanoparticles.

### 3.6. In Vitro Cytotoxicity Test

**Figure 3:**
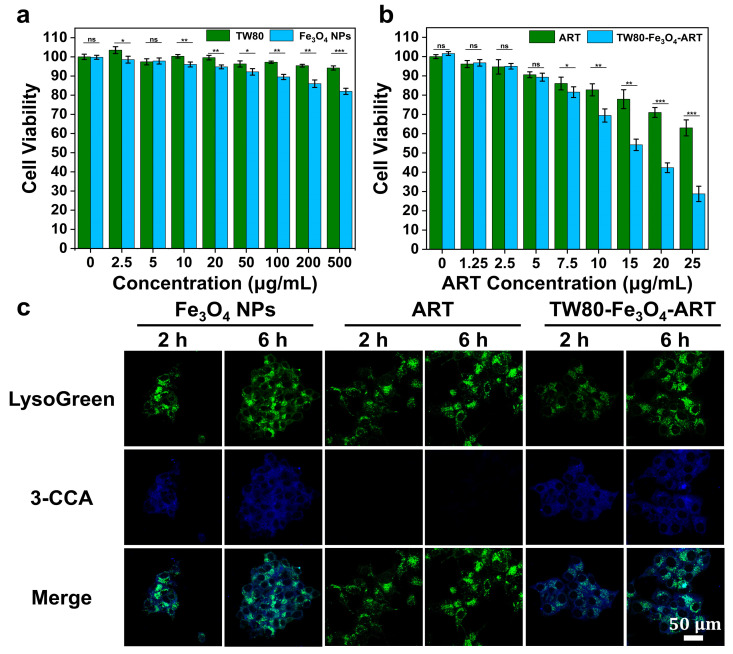
(**a**) Cell viability of MCF-7 cells after incubation with ART (n = 3). (**b**) Cell viability of MCF-7 cells after incubation with Fe_3_O_4_ NPs or TW80-Fe_3_O_4_-ART micelles (n = 3). (**c**) The blue fluorescence was generated from the reaction between intracellular ·OH and 3-CCA. Blue: 3-CCA, green: lysogreen. The bar graphs represent mean ± SD. ns, not significant, * *p* < 0.05, ** *p* < 0.01, *** *p* < 0.001.

The cytotoxicity of TW80, ART, Fe_3_O_4_ NPs, and TW80-Fe_3_O_4_-ART micelles was assessed by the MTT method. In Figure 3a,b, Free artemisinin and Fe_3_O_4_ NPs exhibited limited cytotoxicity against MCF-7 cells cultured for 24 h, even at high concentrations. ART was toxic to MCF-7 cells, and the cell viability was 63.3% when the ART concentration was 25 μg/mL. In contrast, the cellular viability of the TW80-Fe_3_O_4_-ART micelle assembly declined to 28.8%. Furthermore, within the concentration range investigated in our study, TW80 exhibited negligible cytotoxicity.

### 3.7. Intracellular ·OH Detection

The generation of ·OH is commonly regarded as one of the reasons for the toxicity associated with ultra-small Fe_3_O_4_ NPs. The generation of ·OH was studied using 3-CCA as a probe. As shown in Figure 3c, fluorescence was observed in Fe_3_O_4_ NPs and TW80-Fe_3_O_4_-ART micelles treated cells, indicating the generation of ·OH. Meanwhile, the fluorescence of 3-CCA was not overlapped with that of lysogreen, suggesting the ·OH was not generated in lysosomes.

### 3.8. Tissue Distribution Studies

**Figure 4:**
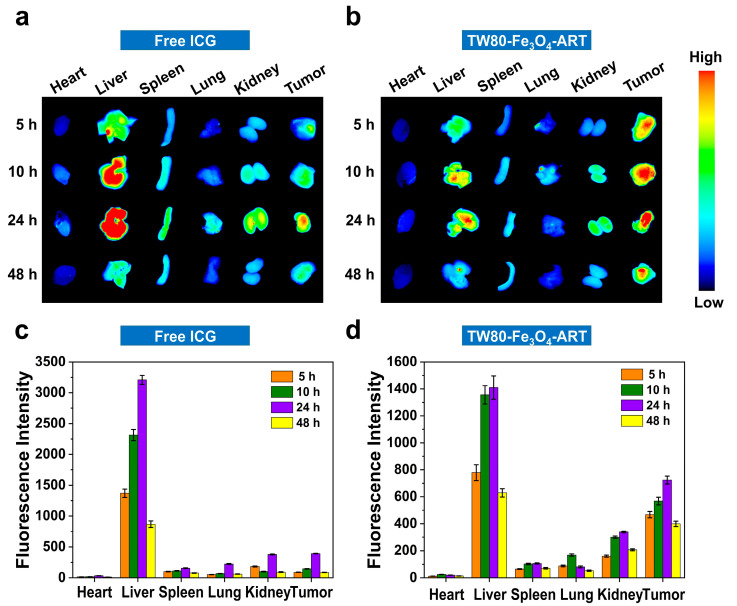
Ex vivo fluorescence images of major organs and tumors were captured at various time points following intravenous injection of (**a**) Free ICG and (**b**) ICG-labeled TW80-Fe_3_O_4_-ART micelles. (**c**,**d**) Quantitative analysis of ICG fluorescence intensity (n = 6). The bar graphs represent mean ± SD.

To study the biodistribution of the TW80-Fe_3_O_4_-ART, the micelles were labeled with indocyanine green (ICG). As depicted in Figure 4a,c, free ICG predominantly accumulated in the liver. Fluorescence in the tumor was weak, reaching the maximum at 24 h. 48 h later, the fluorescence intensity markedly decreased, indicating the excretion of ICG from the body. By contrast, TW80-Fe_3_O_4_-ART micelles showed high accumulation in tumors and retention over 48 h (Figure 4b,d).

### 3.9. In Vivo Therapy

**Figure 5:**
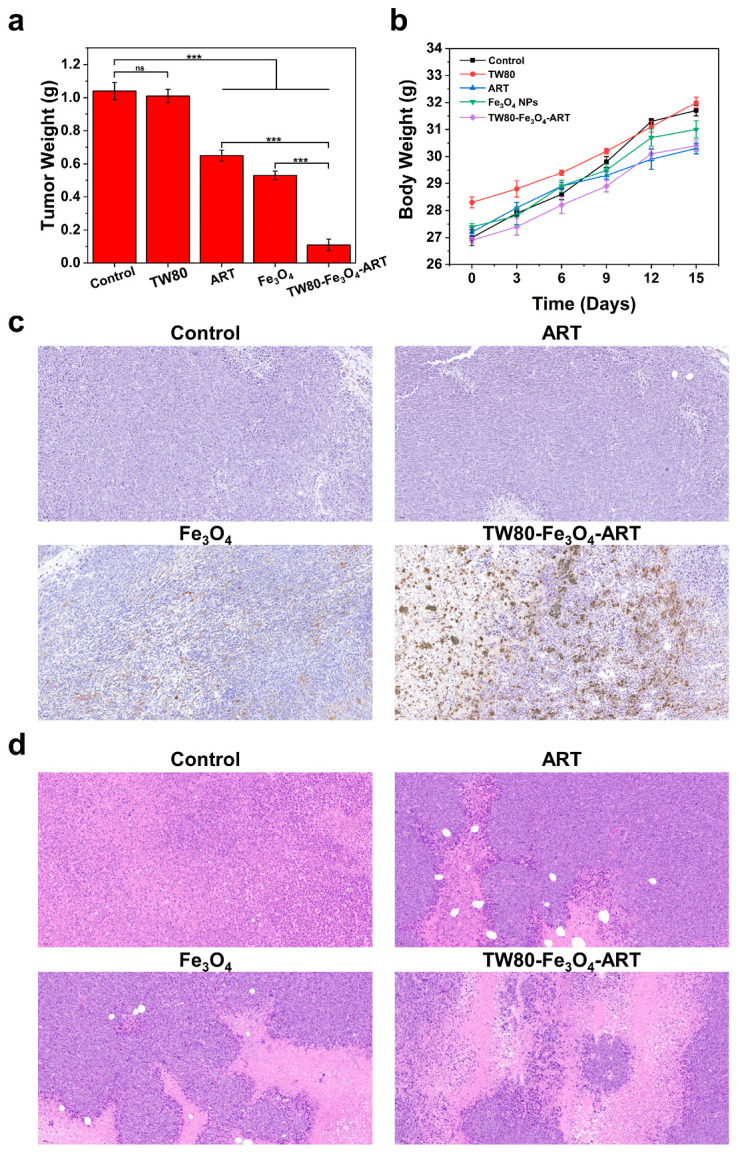
(**a**) The average tumor weights of each group were measured at the end of treatment (n = 6). (**b**) The mice’s body weight over the course of the study (n = 6). (**c**) Prussian blue staining of tumors in mice from different treatment groups. (**d**) Histological images of tumor tissues stained with H&E following various treatments. The bar graph represents mean ± SD. ns, not significant, *** *p* < 0.001.

In vivo anti-tumor experiments were performed to investigate the therapeutic efficacy of TW80-Fe_3_O_4_-ART micelles. As illustrated in Figure 5a, ART and Fe_3_O_4_ could obviously inhibit the growth of tumors, showing inhibition rates of 38% and 46%, respectively. More significant inhibition was observed in the TW80-Fe_3_O_4_-ART group; the inhibition rates reached 85%. During the therapy, the body weight was monitored (Figure 5b). There were no significant differences in body weight among the various groups of mice. The distribution of TW80-Fe_3_O_4_-ART micelles within the tumor was studied by Perls-DAB staining, which stained the iron (Fe) brown. As shown in Figure 5c, although TW80-Fe_3_O_4_-ART micelles and Fe_3_O_4_ NPs were injected at the same dosage, significantly higher iron (Fe) concertation was observed in micelles, indicating a better targeting effect. We also noticed that the distribution of iron (Fe) in the tumor was uniform, which was probably due to the disassembly of the micelles. H&E analysis confirmed the favorable therapeutic effect of TW80-Fe_3_O_4_-ART micelles (Figure 5d) that TW80-Fe_3_O_4_-ART micelles exhibited typical histopathological damage, while only a portion of the tumor was damaged by Fe_3_O_4_ and ART. After the treatment, H&E staining of major organs was performed (Figure S1). No significant damage was observed in the organs between TW80-Fe_3_O_4_-ART micelles and the control group, implying the good biocompatibility and safety of micelles.

## 4. Discussion

The anticancer mechanisms of ART include various processes such as apoptosis, necrosis, inhibition of tumor angiogenesis, and DNA damage. However, notably, ART serves as an inducer of ferroptosis, representing a pivotal aspect of its action. It was widely recognized that Fe^2+^ stands out as the most potent activator of ART [30]. Consequently, iron-based inorganic and inorganic/organic hybrid nanoparticles were regarded as promising candidates for the delivery and controlled activation of ART. These nanoparticles typically comprise a blend of Fe (II) and Fe (III) and hold the potential to release active Fe^2+^ within the mildly acidic milieu of tumor tissues and acidic intracellular compartments, thereby triggering the activation of ART. Therefore, we employed TW80 to achieve co-delivery of Fe_3_O_4_ NPs and ART, aiming to induce cellular ferroptosis and facilitate ART activation, thereby achieving synergistic tumor therapy. Furthermore, ART is easily accessible and economically viable. It exhibits selective cytotoxicity towards tumor cells while sparing normal cells from substantial damage [31]. Currently, some researchers have also employed nanoparticles and exosomes as tools to induce ferroptosis in tumor cells [32]. For instance, Chen et al. [33] developed carrier-free Fe^3+^-ART coordinated nanoparticles, offering enhanced efficacy in cancer treatment. In addition, Fei et al. [34] achieved similar outcomes by synthesizing nano missiles carrying DHA, facilitating ROS generation and GSH depletion, thereby efficiently targeting and killing tumor cells. Multiple studies have demonstrated that ART serves as a potent inducer, driving ferroptosis in tumor cells and effectively reducing tumor size [35,36].

The synthesized iron oxide nanoparticles bore similarities to the IONP 4 synthesized by Hauksdóttir et al. [37], characterized by their ultra-small particle size and hydrophobic properties. Furthermore, the average particle size of the TW80 micelles loaded with ART and Fe_3_O_4_ NPs is smaller compared to that reported by Doost et al. [38] and Sukmawati et al. [39]. The former encapsulated plant-based oregano essential oil (OR) and trans-cinnamaldehyde (TCA) with an average size ranging from 92 to 337 nm, while the latter encapsulated doxorubicin (DOX) and curcumin analogs with an average size of 111.8 nm using TW80. Clearly, the difference in particle size can be attributed to the concentration of TW80 utilized in the preparation of micelles, as well as the molecular weight of the drugs. The Fe^2+^ within the tumor can react with the peroxide bridge structure (-O-O-) of ART, generating free radicals or electrophilic compounds for cancer treatment. Therefore, the anticancer efficacy of ART is positively correlated with the Fe^2+^ content at the target site. We conducted studies on the ability of TW80-Fe_3_O_4_-ART micelles to release Fe^2+^ and generate ROS. These results indicated that in a weak acid environment, Fe_3_O_4_ NPs undergo acid hydrolysis to form Fe^2+^, thus providing a possibility for the generation of the Fenton reaction. Furthermore, the release of Fe^2+^ indeed enhanced the levels of ROS. After 6 h of incubation, the cumulative release rate of ART was higher in acidic environments. This result may be attributed to the exposure of Fe_3_O_4_ NPs over time, which promotes their dissociation and thus facilitates a significant release of ART from the pores. As a result, ART can be loaded onto Fe_3_O_4_ NPs and effectively protected by TW80, thereby preventing premature release during circulation in the body. However, when TW80-Fe_3_O_4_-ART reaches the tumor site (weakly acidic environment), it induces the degradation of Fe_3_O_4_ NPs, thereby hastening drug release.

These results obtained by detecting ROS levels in cells from different treatment groups indicated the synergistic effect of ART and Fe_3_O_4_ owing to the Fe^2+^ catalyzed cleavage of the endoperoxide bridge and the generation of carbonyl radicals. Even in a hypoxic environment, strong fluorescence could be observed, suggesting the generation of radicals was oxygen-independent. Tian et al. [10] investigated the effects of Fe_3_O_4_ NPs with different particle sizes (2, 4, 10, 100 nm) on inducing ferroptosis in cancer cells. Among these, Fe_3_O_4_ NPs of 2 nm and 4 nm induced the highest levels of ROS production and exhibited the strongest DCF green fluorescence after 6 h of incubation with MCF-7 cells. Although in our study, the green fluorescence generated solely by Fe_3_O_4_ NPs was not as intense as observed by Tian et al., this was primarily due to the lower concentration of our Fe_3_O_4_ NPs (lower by 300 μg/mL). However, the green fluorescence produced by our TW80-Fe_3_O_4_-ART, under both aerobic and hypoxic conditions, was significantly higher than that of their 2 nm Fe_3_O_4_ NPs, strongly indicating that co-delivery of Fe_3_O_4_ NPs and ART can markedly enhance intracellular ROS production, thereby achieving the goal of synergistic tumor therapy. As artemisinin (ART) itself lacks fluorescent moieties, we evaluated the cellular uptake of TW80-Fe_3_O_4_-ART in MCF-7 cells by employing doxorubicin (DOX) labeling, utilizing its intrinsic red fluorescence for laser confocal microscopy analysis. Given the pH dependency of ART and Fe^2+^ release as demonstrated in previous studies, the intracellular localization of TW80-Fe_3_O_4_-ART is pivotal. We stained lysosomes with Lysogreen to indicate the intracellular localization of the micelles. Following a 1-h incubation with TW80-Fe_3_O_4_-ART, specific accumulation within lysosomes was observed. Hence, the micelles have the potential for synchronous release of ART and Fe^2+^ under the acidic stimulation of lysosomes. After 4 h of incubation, we also observed bright red fluorescence in the cell nucleus, indicating the substantial release of DOX following the acid-induced disintegration of nanoparticles. Compared to the β-cyclodextrin nanosponges containing doxorubicin (BNS-DOX, 310 nm) prepared by Argenziano et al. [40], our study also observed rapid nuclear accumulation of free doxorubicin within a remarkably short period (<1 h) upon cellular uptake. However, a notable distinction emerged in our investigation. After a 4-h incubation, notable fluorescence was observed within the cell nucleus. This occurrence is presumably linked to the liberation of free DOX from the Fe_3_O_4_ nanoparticles subsequent to lysosomal degradation. Consequently, DOX accumulates within the nucleus, displaying red fluorescence, a phenomenon not observed with BNS-DOX.

And dose-dependent cytotoxicity was observed in the TW80-Fe_3_O_4_-ART group. Wang et al. [41] synthesized nano-particles known as DHA@FCM, incorporating dihydroartemisinin (DHA) and Fe_3_O_4_, for both tumor treatment and imaging applications. At a drug-loaded nanoparticles concentration of 100 μg/mL, our TW80-Fe_3_O_4_-ART micelles demonstrated approximately 10% higher cytotoxicity against tumor cells compared to DHA@FCM nanoparticles. This could be attributed to a higher release of Fe^2+^ and ART following the internalization of the micelles into the cells. The findings from measuring ·OH inside the cells suggested that the presence of Fe_3_O_4_ NPs is essential for the production of ·OH. This was also the main reason for Fe_3_O_4_ NPs inducing cellular lipid peroxidation, damaging the cell membrane system, thereby leading to the phenomenon of ferroptosis.

ICG is a photosensitizer applicable for human use, with a molecular weight of 775 Da. It exhibits infrared absorption characteristics, with an absorption peak at 790 nm in aqueous solution and negligible absorption in the visible light spectrum. The fluorescence signal of free ICG in the control group mainly accumulates in liver tissue and is minimal in tumors, indicating its poor tumor-targeting ability. Excitingly, TW80-Fe_3_O_4_-ART exhibited stronger fluorescence intensity in the tumor region for a considerably extended period. Within 48 h post-injection, there was a noticeable preferential accumulation in tumors rather than in the liver or other tissues as time progressed. As is well-known, a good drug delivery system should exhibit effective therapeutic outcomes while minimizing toxic side effects. Due to the greater accumulation and internalization of the micelles at the tumor site compared to free ART and free Fe_3_O_4_ NPs, the TW80-Fe_3_O_4_-ART group exhibited a higher tumor suppression rate than the other two groups. The results of H&E staining indicated that TW80-Fe_3_O_4_-ART micelles had good biocompatibility and did not cause noticeable systemic toxicity. Furthermore, throughout the entire experimental process, no significant changes were observed in the food intake, water consumption, or skin color of the tumor-bearing mice, indicating minimal side effects associated with the micelles. These results suggested that the co-delivery system of Fe^2+^ and ART assembled through TW80 held significant promise in the realm of tumor therapy.

## 5. Conclusions

In summary, we successfully prepared Fe_3_O_4_ micelles loaded with ART for cancer therapy in an oxygen-independent manner. The co-delivery of Fe_3_O_4_ and ART to the tumor was expected to generate a synergistic effect that the endoperoxide bridge (-O-O-) of ART was cleaved in the presence of iron ions to produce radicals. Meanwhile, the iron ion catalyzed the Fenton reaction to work with radicals generated by ART, resulting in enhanced ferroptosis of cancer. Significantly increased ROS levels and ·OH generation were observed in the co-delivery group. The TW80-Fe_3_O_4_-ART micelles showed an inhibition rate of over 85% in vivo. This work not only indicated a novel platform for drug delivery but also a potential strategy for enhancing ferroptosis for cancer therapy.

## Data Availability

The data presented in this study are available on request from the corresponding author.

## Supplementary Materials

The following supporting information can be downloaded at: https://www.mdpi.com/article/10.3390/pharmaceutics16050639/s1, Figure S1: H&E stained images of major organs of mice after different treatments.
