# Supplementary material for: Tween 80 Micelles Loaded with Fe3O4 Nanoparticles and Artemisinin for Combined Oxygen-Independent Ferroptosis Therapy of Cancer
Source: Pharmaceutics. 2024 May 9;16(5):639. doi: 10.3390/pharmaceutics16050639 (PMC11124998; doi:10.3390/pharmaceutics16050639)
Supplement: Supplementary file 1 [file pharmaceutics-16-00639-s001.zip › pharmaceutics-2983823-supplementary.pdf]

Supporting information

Tween 80 micelles loaded with Fe_3O_4 Nanoparticles and Artemisinin for combined oxygen-independent ferroptosis therapy of cancer

Junming Cui [1,2], Xinxi Cai [1,2], Rui Qian [1,2], Lin Wu [2,*], Xueyong Qi [1], Jin Cao[1] and Song Shen[1,*]

[1] *College of Pharmaceutical Sciences, Jiangsu University, Zhenjiang, Jiangsu 212013, China*

[2] *Affiliated Hospital of Jiangsu University, Zhenjiang 212001, China*

* Corresponding authors:

E-mail address: jsdx.shensong@163.com (S. Shen); linmeimei1983@163.com (L. Wu)

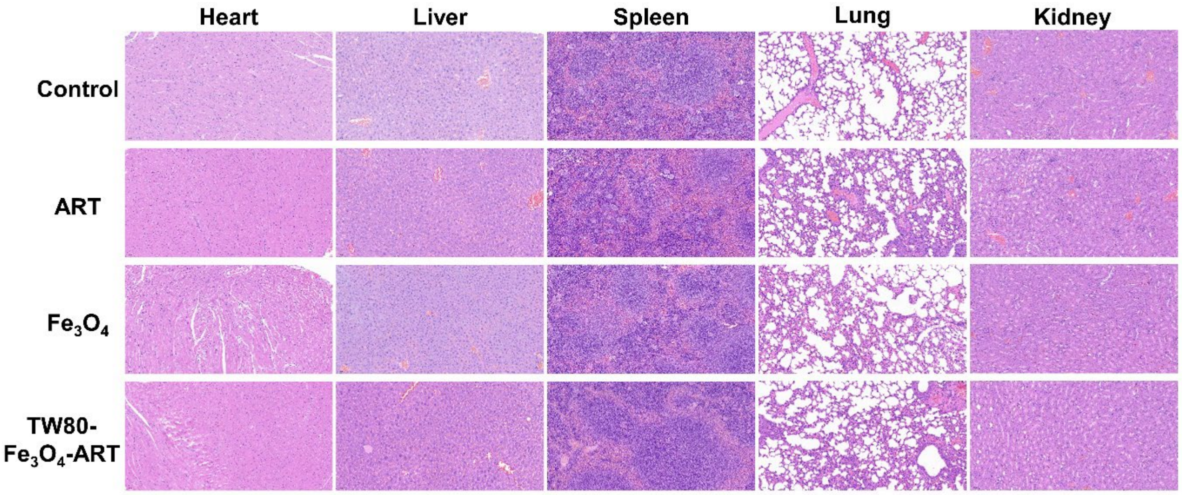

Figure S1. H&E stained images of major organs of mice after different treatments.
